# Highlight selection of radiochemistry and radiopharmacy developments by editorial board

**DOI:** 10.1186/s41181-022-00162-3

**Published:** 2022-04-26

**Authors:** Emerson Bernardes, Peter Caravan, R. Michael van Dam, Winnie Deuther-Conrad, Beverley Ellis, Shozo Furumoto, Benjamin Guillet, Ya-Yao Huang, Hongmei Jia, Peter Laverman, Zijing Li, Zhaofei Liu, Filippo Lodi, Yubin Miao, Lars Perk, Ralf Schirrmacher, Johnny Vercoullie, Hua Yang, Min Yang, Xing Yang, Junbo Zhang, Ming-Rong Zhang, Hua Zhu

**Affiliations:** 1 IPEN, São Paulo, Brazil; 2 Massuchusetts General Hospital, Harvard University, Cambridge, USA; 3 University of California, Los Angeles, Los Angeles, USA; 4 Helmholtz-Zentrum Dresden-Rossendorf (HZDR), Leipzig, Germany; 5 Manchester University NHS Foundation Trust, Manchester, UK; 6 CYRIC, Tohoku University, Sendai, Japan; 7 CERIMED, C2VN, Aix-Marseille Univ, Marseille, France; 8 National Taiwan University College of Medicine, Taipei, Taiwan; 9 Beijing Normal University, Beijing, China; 10 Xiamen University, Xiamen, China; 11 Peking University, Beijing, China; 12 S. Orsola-Malpighi Hospital, Bologna, Italy; 13 University of Colorado, Denver, USA; 14 Radboud University Medical Center, Nijmegen, The Netherlands; 15 University of Alberta, Edmonton, Canada; 16 University of Tours, Tours, France; 17 TRIUMF, Vancouver, Canada; 18 Jiangsu Institute of Nuclear Medicine, Wuxi, Jiangsu, People’s Republic of China; 19 Peking University First Hospital, Beijing, China; 20 NIRS, Chiba, Japan; 21 Peking University Cancer Hospital, Beijing, China; 22 Helmholtz-Zentrum Dresden-Rossendorf, Dresden, Germany

**Keywords:** Highlights, Radiopharmacy, Radiochemistry, Review

## Abstract

**Background:**

The Editorial Board of EJNMMI Radiopharmacy and Chemistry releases a biyearly highlight commentary to update the readership on trends in the field of radiopharmaceutical development.

**Results:**

This commentary of highlights has resulted in 23 different topics selected by each member of the Editorial Board addressing a variety of aspects ranging from novel radiochemistry to first in man application of novel radiopharmaceuticals and also a contribution in relation to MRI-agents is included.

**Conclusion:**

Trends in (radio)chemistry and radiopharmacy are highlighted demonstrating the progress in the research field being the scope of EJNMMI Radiopharmacy and Chemistry.

## Background

Several members of the Editorial Board have selected a highlight article that has appeared in the radiochemistry, radiopharmacy and imaging agent literature during the period July-December 2021. The aim of this collaborative initiative is to create a biyearly overview for the readers summarizing the latest trends in the field.

## Main text

### [Sc-F]: a fresh pair of eyes in nuclear medicine

#### By Emerson Bernardes

In the last decade, nuclear medicine has witnessed several scientific advances regarding radioactive labeling methods involving PET tracers. Most of these advances have focused on the radionuclide fluorine-18 because of its favorable properties for PET imaging. However, ^18^F does not have a true theranostic pair. Recently, an alternative [^18^F] radiolabeling method was brilliantly validated (Whetter et al. 2021), showing for the first time that the formation of the ternary [^18^F][Sc-F] complex is viable and inert to defluorination in vivo. In addition, [^18^F][Sc-F] complexes are ideally suited to the incorporation of ^47^Sc, which renders the [^18^F]/[^47^Sc] isotope pair an unusual, but viable theranostic option with prospective clinical utility.

Although the difficulty to produce large, clinically relevant quantities of ^47^Sc remains a key factor, research teams across the world have been pursuing their work on finding ways to overcome these limitations. Therefore, the growing interest on the medical applications of ^47^Sc, which has been suggested as an alternative radionuclide to ^177^Lu, and also the fact that ^18^F is the “radionuclide of choice” for PET imaging, should warrant for further research on the ^18^F/^47^Sc isotope pair for years to come.

### Radioiodine/^211^At-labeled neopentyl glycol: a novel scaffold for radiotheranostic agents

#### By Shozo Furumoto

**Fig. 1:**
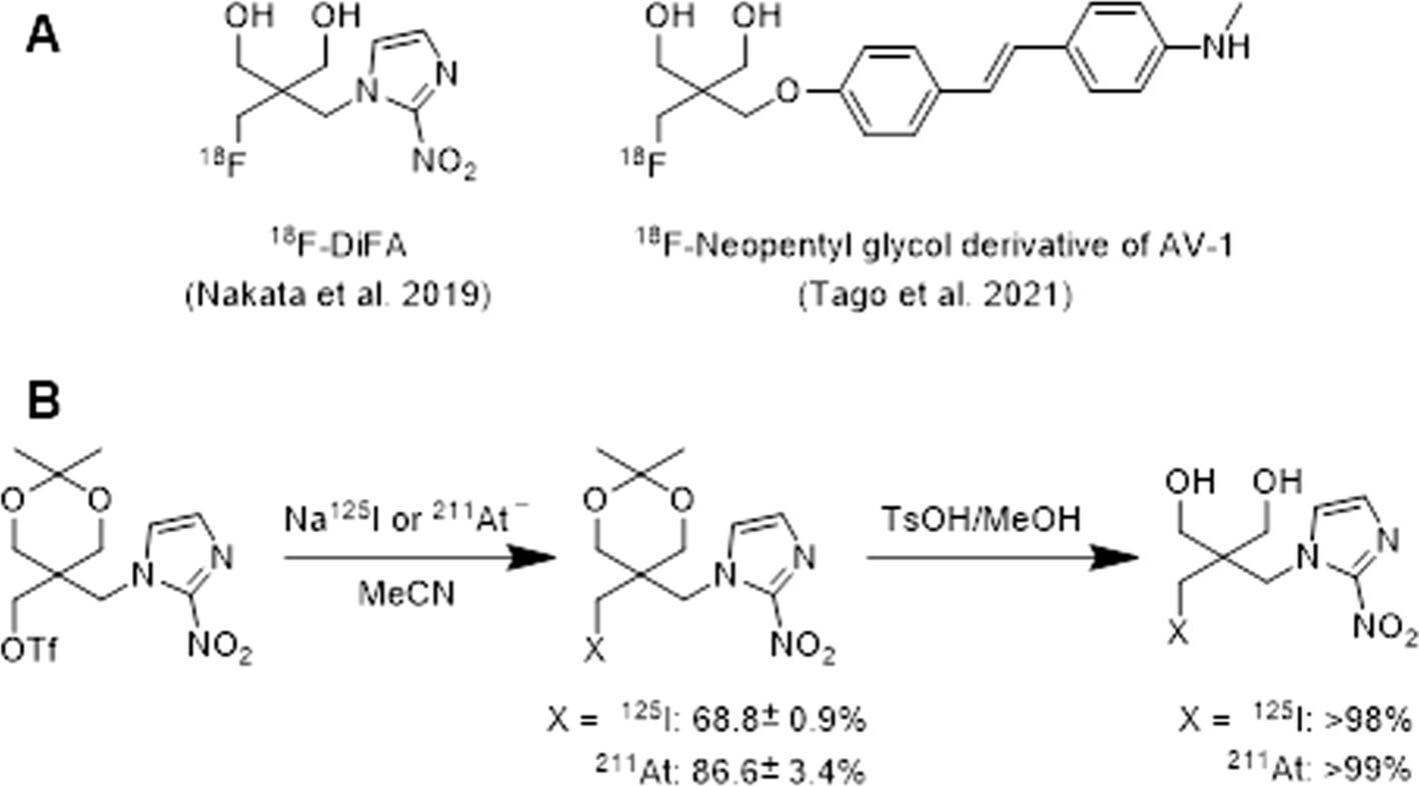
Structures of ^18^F-labeled neopentyl glycol derivatives (**A**) and a scheme for radiosynthesis of ^125^I/^211^At-labeled neopentyl glycol derivatives (**B**)

Astatine-211 is one of the most promising α-emitters for targeted α-therapy due to its desirable physical properties such as half-life of 7.2 h, α-particle emission rate of 100%, and lack of long-lived daughter α-emitter causing side effects. Chemical properties of astatine which belongs to the halogen group are similar to those of iodine. Thus, ^211^At-labeled compounds can be synthesized under conditions almost the same as radioiodination. These features lead us to believe that radioiodine (^123^I, ^124^I) and ^211^At are an attractive combination of radioisotopes for theranostics. However, to achieve such radiotheranostics, it is necessary to establish a reliable radiohalogenation method for preparing metabolically stable tracers because radioiodine/^211^At-labeled compounds tend to exhibit metabolic dehalogenation in vivo. Recently, Suzuki et al. reported that neopentyl glycol is a promising scaffold for developing radiotherapeutic systems using radioiodine and ^211^At (Suzuki et al. 2021). Their rationale for adopting the skeleton for radiolabeling was based on the fact that ^18^F-labeled neopentyl glycol derivatives (Fig. 1A) are stable against defluorination metabolism in vivo (Nakata et al. 2019; Tago et al. 2021). They designed nitroimidazole derivatives with a ^125^I/^211^At-labeled neopentyl glycol scaffold as an analog of ^18^F-DiFA. The ^125^I/^211^At-labeled products were synthesized in high radiochemical yields from the corresponding triflate precursors by reacting with Na^125^I/^211^At^−^ (Fig. 1B). In vitro assays using mouse and human liver microsomes demonstrated that the ^125^I/^211^At-labeled derivatives are resistant to CYP-mediated metabolism. In vivo administration of the derivatives resulted in low radioactivity accumulation in the stomach and neck (thyroid) even 24 h post-injection. Additionally, no metabolite ^125^I^−^ or ^211^At^−^ was observed in the urine samples. These results indicate that the ^125^I/^211^At-labeled neopentyl glycol structure is highly stable against in vivo dehalogenation. Moreover, the radioiodination and astatination of neopentyl glycol can be achieved with mild reaction conditions and simple manipulations, making it easy to automate the synthesis system. The findings of this study could provide a new practical approach to the development of radiopharmaceuticals for radiotheranostics using radioiodine and ^211^At.

### The comparison of bifunctional chelators between NODAGA and DOTA using ^68^Ga-LM3

#### By Hua Zhu

*p*-Cl-Phe-cyclo(D-Cys-Tyr-D-4-amino-Phe(carbamoyl)-Lys-ThrCys)D-Tyr-NH2 (LM3) is a novel SSTR antagonist. It can be coupled with different chelators (NODAGA, DOTA) and the radiometal ^68^Ga, resulting in a binding affinity to SSTR2, with a 50% inhibitory concentration of 1.3 and 12.5 nmol/L respectively. Recently, both ^68^Ga-NODAGA-LM3 and ^68^Ga-DOTA-LM3 were investigated for the comparison of safety, biodistribution, dosimetry (phase I), and diagnostic efficacy (Zhu et al. 2021).

This paper describes the first clinical study to evaluate these two tracers in patients with NETs. The preliminary results show favorable biodistribution and dosimetry features, and both tracers were well tolerated in most patients. Both tracers display high tumor uptake, and good tumor retention, resulting in high imaging contrast.

As can be concluded from the paper, the lower background of ^68^Ga-DOTA-LM3 resulted into significantly higher tumor-to-kidney and tumor-to-liver ratios. The simpler the better.

### Towards the stable chelation of radium for biomedical applications with an 18-membered macrocyclic ligand

#### By Xing Yang

^223^Ra has attracted widespread interest in the field of cancer treatment because of its clinical efficacy, safety and availability as an α particle-emitting radionuclide. Compared with β rays, α rays have higher linear energy and shorter tissue penetration distance, which allows for more effective and selective killing of target cells. [^223^Ra]RaCl_2_ has been approved for clinical use to treat bone metastases of castrate-resistant prostate cancer, where [^223^Ra]Ra^2+^ can accumulate due to its intrinsic biodistribution properties. To extend its applications and direct [^223^Ra]Ra^2+^ to non-osseous disease sites, the development of a stable chelator is necessary for conjugation to receptor-targeting moieties. However, the efforts so far have been unsuccessful, making it a challenging issue for the field.

Diane S. Abou reported a [^223^Ra]Ra^2+^ complex, [^223^Ra]Ra(macropa), with the 18-membered bispicolinate diazacrown macrocyclic chelator macropa (Abou et al. 2021), which also demonstrated high affinity for Ba^2+^. Quantitative labeling of macropa with [^223^Ra]Ra^2+^ could be accomplished within 5 min at room temperature with a radiolabeling efficiency of > 95%. The macropa conjugate [^223^Ra][Ra(macropa-balanine)] also showed good stability both in vitro and in vivo, with lower kidney uptake and faster renal clearance than [^223^Ra]RaCl_2_. Finally, the authors conjugated macropa to the prostate-specific membrane antigen-targeting peptide DUPA and successively radiolabeled [^223^Ra]Ra(macropa)-DUPA. Loss of stability was observed for in vivo experiments, indicating targeting vectors could affect metal-chelate stabilities. Nevertheless, the success of stably chelation was proved for the first time to deliver [^223^Ra]Ra^2+^ to lesions outside of the bone, which might expand the therapeutic utility of [^223^Ra]Ra^2+^ and open the path for receptor-targeted α particle radiotherapy with ^223^Ra.

### New insights into in vivo tracking of chimeric antigen receptor T cells therapy: ICOS-immunoPET imaging

#### By Min Yang

Chimeric antigen receptor (CAR) T cell therapies have made unprecedented advances in the treatment of hematological malignancies. Current immunomonitoring of CAR T cells in the clinic relies primarily on their quantification in the peripheral blood and bone marrow, an invasive assay that is insufficient to quantify their biodistribution and activation status in tissues. Non-invasive PET imaging tracking of CAR T cells is a promising approach to provide spatial, temporal and functional information. Reported strategies rely on the incorporation of reporter transgenes or ex vivo labeling, which greatly limit the clinical application of CAR T-cell molecular imaging. The publication by Volpe et al., demonstrated that immunoPET targeting Inducible T-cell COStimulator (ICOS or CD278), a costimulatory molecule upregulated during T-cell activation, enables in vivo imaging of activated CAR T cells at the tumor site (Volpe et al. 2021). Early timepoint studies (48 h after tracer administration on day 5 after CAR T cells administration) demonstrated the ability of tracking the CD19 CAR T cells to migrate to their target tissue, bone marrow bearing CD19 antigen on infiltrating B-cell lymphoma cells. This molecular imaging approach targeting an endogenous biomarker does not require additional reporter genes or ex vivo labeling. Therefore, it is potentially applicable for the study of dynamic proliferation kinetics of any commercially available and investigational stage CAR T-cell products for any targets in a clinical setting, with promising clinical translation prospects.

### [^18^F]AlF-based radiopharmaceuticals as diagnostic counterpart in FAP-targeted radiotheranostics

#### By Winnie Deuther-Conrad

The plasma membrane form of the serine protease fibroblast activation protein (FAP) is involved in the degradation of extracellular matrix (ECM) proteins. Due to the complex interaction between the resident cells and the ECM, integrity, structure and composition of the ECM are of high relevance for (patho)physiological processes such as differentiation, proliferation, and migration. A selective overexpression of FAP in the stroma of epithelial tumors and malignancies of bone and soft tissues contributes to the invasiveness and progression of cancer. According to the relevance of FAP as prognostic and predictive biomarker, FAP-targeted molecules such as small molecule inhibitors (FAPIs) are of interest as radioactive theranostic drugs used to diagnose and treat the primary and any metastatic tumors.

By applying the aluminium-[^18^F]fluoride radiolabelling method, [^18^F]AlF-FAPI-74, a new radiolabelled FAPI was developed with the potential to improve the diagnostic phase of theranostics by increasing the scalability in comparison to the ^68^Ga-labelled FAPIs in terms of e.g. supply of the radionuclide and delivery of the radiopharmaceutical (Lindner et al. 2021). The NOTA-based [^18^F]AlF-FAPI-74 has been obtained under mild reaction conditions and with low precursor consumption with high radiochemical yield. The good tumour accumulation and contrast along with a favourable clearance and renal excretion observed in animal studies has been confirmed by the results of the first-in-human investigation in one patient with NSCLC. The implementation of the [^18^F]AlF radiolabelling method in the development of FAP-targeted theranostics reflects the relevance of this radiochemical approach for the benefit of cancer patients.

### Potential of producing ^155^ Tb at small medical cyclotrons

#### By Hua Yang

Terbium (Tb) has four medical relevant isotopes, ^161^Tb (β-therapy), ^155^Tb (SPECT), ^152^Tb (PET) and ^149^ Tb (α-therapy, PET), covering all major nuclear medicine modalities and providing unique opportunities to develop diagnostic and therapeutic radiopharmaceuticals with chemically identical compounds. However, the availabilities of the four Tb isotopes are not equal – while ^161^ Tb is produced at high quantity at reactors, ^155^ Tb, ^152^ Tb and ^149^Tb were only produced at cyclotron mass separation stations which only CERN and TRIUMF have, until a recent report on the production ^155^ Tb at lower energies from enriched targets (Favaretto et al. 2021). This report explored the ^156^Gd(p,2n)^155^ Tb reaction at ~ 23 MeV, and ^155^Gd(p,n)^155^ Tb reaction at ~ 10 MeV and 18 MeV. Enriched Ga_2_O_3_ was used as target material, and target design was described. The irradiated targets were purified and it was discovered although ^156^Gd(p,2n)^155^ Tb reaction produced more activity (1.7 GBq) compared to ^155^Gd(p,n)^155^ Tb reaction (200 MBq), ^155^Gd(p,n)^155^ Tb product is superior in radionuclide purity particularly in terms of other Tb isotopes. ^155^ Tb produced was used to label DOTATATE, and high molar activity was achieved (up to 100 MBq/nmol). The in vivo imaging in AR42J tumor bearing mice at 1, 4 and 24 h was performed, and the images are comparable to previous reports using ^155^ Tb produced from a mass separation station. This report paves the ground to improve the availability of ^155^ Tb for a broader research community.

### Target manufacturing by spark plasma sintering for efficient ^89^Zr production

#### By Lars Perk

S. Cisternino and colleagues developed an alternative novel approach for the production of zirconium-89 (^89^Zr) by using a solid target manufactured by spark plasma sintering (SPS) (Cisternino et al. 2022). The use of ^89^Zr for radiolabeling antibodies and antibody fragments used in research and clinical development has boomed over the last ten years. The most common ^89^Zr production route is through the ^89^Y(p,n)^89^Zr nuclear reaction using a target, mostly foils or sputtered targets, composed of 100% naturally abundant and readily available yttrium-89 material. In this study, the authors evaluated the use of SPS technique to tightly bind a thin 150 µm yttrium disc to a niobium backing plate. Niobium is an excellent option due to its high melting temperature, sufficient thermal conductivity and chemical inertness. The average yield of ^89^Zr produced using SPS targets was approx. 14.12 MBq/µAh (up to 3.5 GBq ^89^Zr after purification), which is comparable with data obtained using standard yttrium foil targets (Synowiecki et al. 2018). The effective molar activity of approx. 140 GBq/µmol is higher as compared to the use of yttrium foils. Other parameters, like the radionuclidic and radiochemical purity, are similar to previous published data using established productions routes. In conclusion, SPS-made yttrium targets might be an attractive and cost-effective alternative. It would be interesting to see what will be the effect of irradiating a thinner layer of yttrium and/or with an angle of a few degrees between the incident beam and material layer.

### Inorganic radiopharmaceutical chemistry of oxine

#### By Peter Laverman

Recently, the radiopharmaceutical chemistry of the oxine molecule was extensively discussed in all its aspects (Southcott and Orivig 2021). Radiolabeled cells have been used for over decades and the first use of [^111^In]In-oxine labeled leukocytes has been described in the 1970s (McAfee and Thakur 1976) and in 1985 the compound was approved by the FDA. Although ^99m^Tc-HMPAO was, and still is, widely used as cell labeling agent, the use of oxine is reviving with the increased interest in tracking of immune cell (subsets) which generally requires later imaging time-points. While oxine initially has been investigated as a cell and nanomedicine labeling agent for ^111^In, also ^68^Ga has been proposed, however its short half-life limited wide-spread application. With an increasing demand for nanomedicine and cell tracking with PET imaging, mainly due to its higher sensitivity, the use of oxine in combination with ^89^Zr for PET imaging has been demonstrated (Ferris et al. 2014; Sato et al. 2015). Although initially radiochemically challenging due to the multi-step synthesis, the recent development of a labeling kit will most likely further speed up the widespread use of ^89^Zr-oxine cell labeling (Man et al. 2020).

### Comparison of [^68^Ga]Ga-FAPI-04 and [^18^F]FDG for the detection of primary and metastatic lesions in patients with gastric cancer: a bicentric retrospective study

#### By Zijing Li

Up to 53% of primary gastric cancers are not avid for [^18^F]FDG, whose uptake is strongly associated with the histological type and size of the tumour (Kaneko et al. 2015; Herrmann et al. 2007). The low sensitivity for the detection of lymph node metastases and peritoneal metastases limited the use of [^18^F]FDG for determining the clinical stage of gastric cancer (Herrmann et al. 2007). Previous studies have shown that fibroblast activation protein (FAP) is overexpressed in the cancer-associated fibroblasts (CAFs) of gastric cancer and plays an important role in the invasion and migration of gastric carcinomas (Wang et al. 2013; Zhi et al. 2010). With high affinity and suitable kinetics, [^68^Ga]Ga-FAPI-04 has achieved good outcomes with regard to the diagnosis and staging of several tumor types (Kratochwil et al. 2019; Chen et al. 2021a, b; Koerber et al. 2020).

Donglang Jiang et al. examined 38 patients confirmed by pathological biopsy (Jiang et al. 2021). All of the participants underwent [^68^Ga]Ga-FAPI-04 and [^18^F]FDG imaging by PET/CT or PET/MR and the SUV_max_ was calculated. For the detection of primary gastric cancer, the sensitivities of [^68^Ga]Ga-FAPI-04 PET and [^18^F]FDG PET were 100% (38/38) and 82% (31/38), respectively (*P* = 0.016). For the detection of metastatic lesions, the sensitivities of [^68^Ga]Ga-FAPI-04 PET and [^18^F]FDG PET in 10 patients with regional lymph node metastasis and distant metastasis were 6/10 and 5/10, respectively. It was concluded that [^68^Ga]Ga-FAPI-04 PET is superior to [^18^F]FDG PET for the detection of primary gastric cancers especially for tumors less than 4 cm in size and [^68^Ga]Ga-FAPI-04 PET could provide insight into the degree of tumor invasion in gastric cancer.

This study was the first time to report the potential advantage of [^68^Ga]Ga-FAPI-04 in diagnosing gastric cancer and the difference between [^68^Ga]Ga-FAPI-04 and [^18^F]FDG. However, it is necessary to evaluate the N (metastatic lymph nodes) and M (distant metastases) staging value of [^68^Ga]Ga-FAPI-04 in gastric cancer in more cases of signet ring cell carcinoma and in a larger cohort. Whether [^68^Ga]Ga-FAPI-04 PET has advantages for the detection of small gastric cancers is still not determined.

### A simplified method of gaseous [^18^F]Tryflyl fluoride generation would facilitate one-pot or in-loop production of ^18^F-radiopharmaceuticals without conventional azeotropic drying process.

#### By Ya-Yao Huang

Azeotropic drying with acetonitrile has been a standard procedure to obtain reactive [^18^F]fluoride in nucleophilic radiofluorination of most of ^18^F-radiopharmaceuticals. However, such procedure has many drawbacks such as solvent degradation or nonspecific vessel absorption resulting from overheating [^18^F]fluoride/K_2_CO_3_/ Kryptofix 222(K_222_). Thus, a reliable method for generating [^18^F]triflyl fluoride ([^18^F]TfF) as a gaseous source of [^18^F]fluoride was been reported in 2018 (Pees et al. 2018). However, the use of moisture sensitive P_2_O_5_ and three-pot automation will be challenges during such routine ^18^F-radiopharmaceutical production (Pees et al. 2020).

**Fig. 2:**
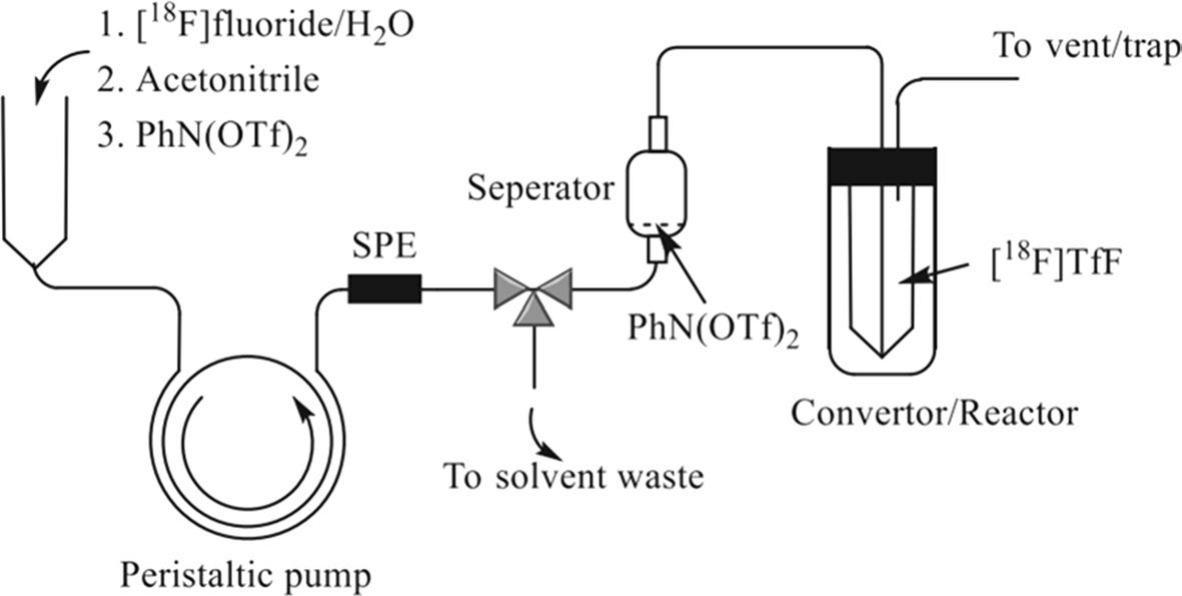
Schematic illustration of the continuous-flow generation process of [^18^F]TfF (With permission from Zhou and Katzenellenbogen (2021) by Copyright Clearance Center’s Rightslink)

Recently, a simple, continuous-flow solid-phase method for in-line [^18^F]TfF generation was developed (Fig. 2) with adjusting the used base and solvent, and well-controlled flow rate (Zhou and Katzenellenbogen 2021). Such process provided a good trapping efficiency of > 90% of [^18^F]TfF in less than 10 min when less K_2_CO_3_ (0.15 mg) in K_2_CO_3_ /K_222_ (1 mg) and flow rate of 3 mL/min were adopted, and further comparable radiofluorination rates were achieved for [^18^F]FDG, [^18^F]4-*FGln* and [^18^F]FluorThanatrace (Zhou et al. 2014). Overall, it would be a promising approach for automated “one-pot” or “in-loop” radiosyntheses of a variety of ^18^F-radiopharmaceuticals without cumbersome azeotropic drying procedure, especially for a range of ^18^F-labelled base-sensitive compounds, such as [^18^F]fluoroform (Yang et al. 2019; Huiban et al. 2013; Pees et al 2021).

### S_N_Ar Radiofluorination with in situ generated [^18^F]Tetramethylammonium fluoride

#### By Ralf Schirrmacher

**Fig. 3:**
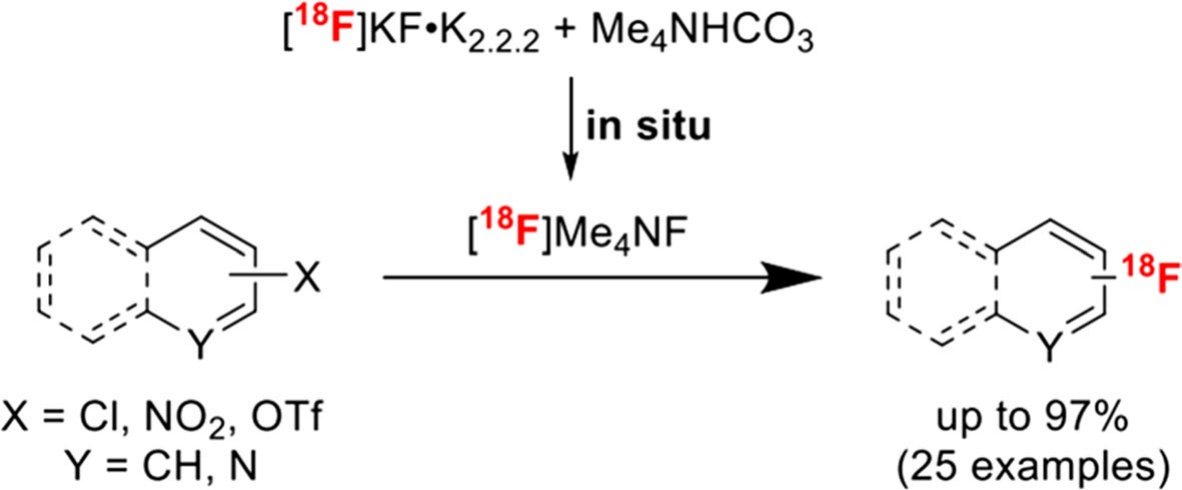
Schematic representation of in situ generation of [^18^F]Me_4_NF and subsequent aromatic fluorination

The in situ generation of the highly reactive ^18^F-fluorination agent [^18^F]tetramethylammonium fluoride ([^18^F]Me_4_NF) constitutes an important refinement of the frequently applied radiolabeling of activated hetero aromatic scaffolds. Fluorinated pyridine and quinolone derivatives are important building blocks in drug development. Hence, their efficient and convenient ^18^F-labeling is important for PET tracer development. A great variety of (hetero)aryl chlorides and triflates as well as nitroarenes have been ^18^F-radiolabeled in good to excellent yields using azeotropically dried [^18^F]KF K_222_, the most commonly applied labeling agent in PET tracer synthesis, and Me_4_NHCO_3_ as an in situ additive to form the strongly nucleophilic fluorination agent [^18^F]Me_4_NF (Fig. 3). This improved synthesis protocol avoids the independent preparation of dry [^18^F]Me_4_NF enabling the rapid and relatively mild ^18^F-fluorination of activated hetero aromatic precursors (Jeong Lee et al. 2021). The simplicity and the potential scope of this methodology was further exemplified by the automated PET tracer synthesis of [^18^F]HQ415, a radiotracer intended to image toxic protein deposits in amyotrophic lateral sclerosis (ALS).

### Use of [^177^Lu]FAPI-46 and [^225^Ac]FAPI-46 in a pancreatic cancer model: in search of the best tracer kinetics and the optimal radionuclide for FAPI radioligand therapy

#### By Filippo Lodi

Fibroblast activation protein (FAP), selectively expressed by cancer-associated fibroblasts (CAFs) in tumor stroma, is considered an interesting target for the detection and treatment of malignant tumors with theranostic tracers. Several small-molecule inhibitors of FAP (FAPI) were developed and labeled with PET radioisotopes and used in clinical diagnostics of various cancers (Kratochwil et al. 2019). However, the clinical value of therapeutic use of FAPI tracers is still to be demonstrated, since reports regarding this application are relatively limited. Moreover, the optimal therapeutic radionuclide needs to be found. Therefore, studies are needed to investigate the best combination of kinetics and physical decay for the possible application of FAPI radioligand therapy. Recently, the therapeutic effects of FAPI-46 with improved tumor retention labeled with a beta (^177^Lu) and an alpha (^225^Ac) emitter in pancreatic cancer models were compared (Liu et al. 2021). The biodistribution and treatment effects were evaluated with different doses injected into PANC-1 xenograft mice. Both tracers showed tumor-suppressive effects: [^177^Lu]FAPI-46 showed mild but more prolonged therapeutic effects as compared to [^225^Ac]FAPI-46, supposedly due to beta emission characteristics. The authors suggested the possible application of alpha and beta therapy targeting FAP for pancreatic cancer. However, since the biological half-life of FAPI is short compared to the long physical half-life of ^177^Lu and ^225^Ac, further evaluations are necessary to find the best combination of fast FAP kinetics and physical decay of the radionuclide: the use of high radioactivity dose with shorter half-life radionuclides (^188^Re or ^211^At) could be a possible strategy to improve treatment effect.

### ImmunoPET of new checkpoint receptor toward precision immunotherapy

#### Zhaofei (Jeff) Liu

Inhibitory therapies targeting new immune checkpoint receptors beyond PD-1/PD-L1 and CTLA-4 have emerged as promising approaches for tumor immunotherapy. However, these therapies rely on the quantification of receptor expression by biopsy followed by immunohistochemistry for patient selection, which is invasive and cannot provide accurate information of the whole tumor or metastatic lesions due to tumor heterogeneity.

ImmunoPET, which takes advantage of both the high specificity of antibodies and the high sensitivity of PET, provides a whole-body, noninvasive, and quantitative method to measure the expression of biomarkers (antigens) in vivo, which would facilitate patient stratification and the rational design of therapy regimens for immune checkpoint blockade. A new development of ^64^Cu- and ^89^Zr-labeled antibodies targeting the T-cell immunoreceptor with Ig and ITIM domains (TIGIT) for immunoPET of TIGIT expression in the tumor microenvironment in tumor-bearing mouse models was reported (Shaffer et al. 2021). Both radiotracers exhibited high immunoreactivity and specificity for TIGIT. In a melanoma mouse model, PET using ^89^Zr-labeled anti-TIGIT antibody can sensitively detect the expression of TIGIT on tumor-infiltrating lymphocytes. Further development and optimization of clinically applicable TIGIT-targeting radiotracers (e.g., radiolabeled humanized antibodies/antibody fragments/nanobodies; and radiolabeled small-molecule motifs, such as peptides and active proteins) and harnessing the power of PET using them would provide valuable information for the stratification of patients for anti-TIGIT immunotherapies, dose optimization, and therapy responses monitoring.

### Fast ion-chelate dissociation rate for in vivo MRI of labile zinc with frequency-specific encodability

#### By Peter Caravan

**Fig. 4:**
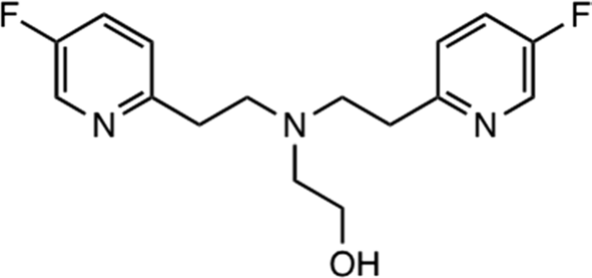
Chemical structure of fluorinated chelate with fluorine substituents in the pyridine rings

The optimization of a chelator containing F-19 atoms for selective sensing of Zn^2+^ in vivo has been described (Tirukoti et al. 2021). When the chelate as shown in Fig. 4 binds to Zn^2+^ the resulting complex has a different F-19 chemical shift. The chelator binds weakly resulting in a fast chemical exchange between chelated and unchelated Zn^2+^. By selective excitation at the F-19 frequency of the complex, they saturate this resonance, and because the system is in fast chemical exchange, the saturation is transferred to the F-19 frequency of the uncoordinated ligand. MRI is an insensitive technique, but they can deliver enough ligand to be detectable (mM concentration). When they excite at the F-19 frequency of the complex, the saturation transfer results in a reduction of signal of the uncoordinated ligand. The Zn^2+^ is exchanging in and out of the ligand over 800 times per second and as a result amplifies this effect on the uncoordinated ligand. By comparing the signal intensity of the ligand with and without saturation of the complex, they can indirectly sense Zn^2+^ at micromolar concentrations that could not be directly detected by MRI. Proof of concept is demonstrated in the brain of a live mouse.

### 5-methyltetrahydrofolate-based conjugates for folate receptor-targeted radionuclide therapy

#### By Beverley Ellis

The folate receptor (FR) is overexpressed in a variety of tumour types and has been identified as a potential target for radionuclide therapy. The targeting of the folate receptor for radionuclide therapy is challenging as there is a relatively low tumour-to-kidney ratio of folate conjugates. The introduction of an albumin-binding entity into the structure of radiofolates has previously been shown to improve tumour-to-kidney ratios (Siwowska et al. 2017).

**Fig. 5:**
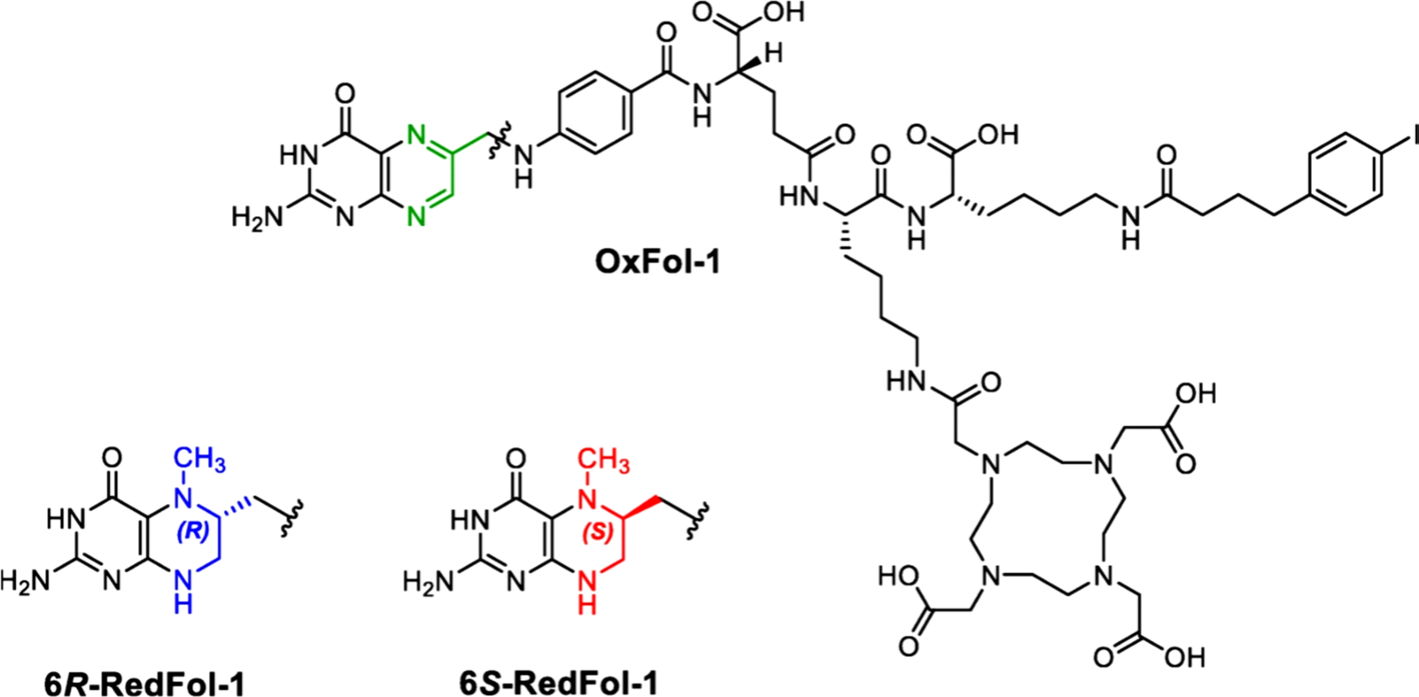
Chemical Structure of Ox-Fol-1 (green), 6R-RedFol-1 (blue), and 6S-RedFol-1 (red). Reproduced for Guzik et al 2021

Recently, an albumin-binding radioconjugates using 5-methyltetrahydrofolate as a targeting agent with the aim of increasing tumour uptake and possibly reducing the renal retention of activity (Guzik et al. 2021). 6*R*-RedFol-1 and 6*S*-RedFol-1 were radiolabelled with lutetium-177 and evaluated in-vitro and in-vivo and their characteristics compared with those of the previously developed [^177^Lu]Lu-OxFol-1 (Fig. 5).

[^177^Lu]Lu-6*R*-RedFol-1 and [^177^Lu]Lu-6*S*-RedFol-1 showed high stability in PBS and human plasma in vitro. A higher in vitro uptake and internalization in FR-positive KB tumour cells was observed with [^177^Lu]Lu-6*R*-RedFol-1 and [^177^Lu]Lu-6*S*-RedFol-1 compared with [^177^Lu]Lu-OxFol-1 and the in vivo results showed a 3–fourfold increased tumour uptake of [^177^Lu]Lu-6*R*-RedFol-1 and [^177^Lu]Lu-6*S*-RedFol-1 in KB tumour-bearing mice. Higher tumour-to-kidney ratios were reported with [^177^Lu]Lu-6*R*-RedFol-1 compared with the other radioconjugates. [^177^Lu]Lu-6*R*-RedFol-1 was also shown to have enhanced therapeutic efficacy compared with [^177^Lu]Lu-OxFol-1. The authors conclude that [^177^Lu]Lu-6*R*-RedFol-1 is a promising radioconjugate for future clinical translation.

### Development of mitochondria-targeted small-molecule dyes for myocardial PET and fluorescence bimodal imaging

#### By Yubin Miao

**Fig. 6:**
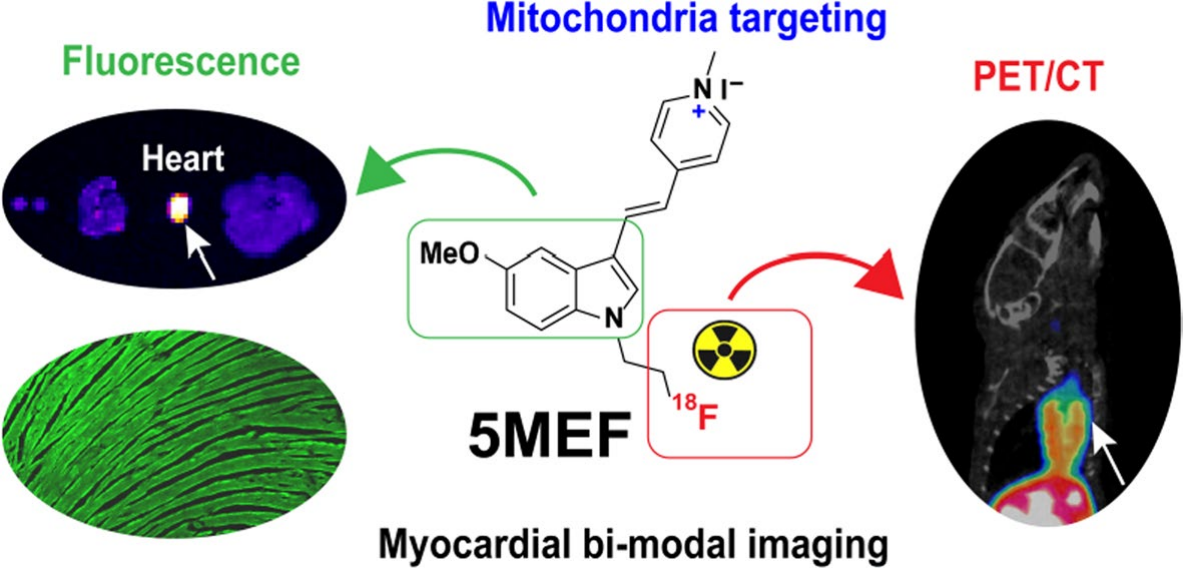
Chemical structure of [^18^F]5MEF and its application in myocardial bimodal imaging. Reprinted with permission from Journal of Medicinal Chemistry (*J Med Chem* 2022; 65: 497–506). Copyright © 2022 American Chemical Society

A novel class of ^18^F-labeled mitochondrial-targeted (*E*)-4-(1*H*-Indol-3-ylvinyl)-*N*-methylpyridinium iodide (F16) compounds was synthesized and evaluated for myocardial positron emission tomography (PET) and fluorescence bimodal imaging (Zheng et al. 2022). F16 is a lipophilic cation which is often used in mitochondria-targeted tumor imaging and treatment. The study design takes advantage of the mitochondrial-targeting and intrinsic fluorescence properties of F16 derivatives, and the substitutions at the indole ring to modify the lipophilicity and cytotoxicity of F16 compounds. In vitro cellular fluorescence imaging revealed that the lead compound 5MEF precisely localized in the mitochondria of cardiomyocytes, whereas the PET imaging of [^18^F]5MEF demonstrated its high heart uptake and heart-to-normal tissue contrast in healthy nude mice (Fig. 6). Overall, this interesting report may encourage and facilitate more research efforts to develop novel mitochondria-targeted myocardial imaging agents in the future.

### Regarding [^18^F]AlF developments and uses: is there a need for ^68^Ga radiopharmaceuticals?

#### By Johnny Vercoullie

Compared to ^68^Ga, ^18^F offers numerous advantages: longer half-life, a more favorable dosimetry for patient, higher availability and quantities of radioactivity produced. On what scientists can influence, the availability and quantities of radioactivity produced have been recently improved with the authorization for clinical application of gallium-68 obtained from cyclotron. Nevertheless, this improvement compared to the use of GMP generators is far to allow the activities produced for nucleophilic [^18^F]fluoride. Thus, the aluminium-[^18^F]fluoride ([^18^F]AlF) offers an interesting alternative to ^68^Ga for the radiolabelling of radiopharmaceuticals bearing chelators. As recently reported (Archibald and Allot 2021) [^18^F]AlF is easy to prepare and can be used in the production of radiopharmaceuticals. Main limitation is the pH restriction as to get the reactive [^18^F]AlF^2+^ species pH has to be in a narrow range of 4–5. As for ^68^Ga various chelators were evaluated (e.g. DTPA, NODAGA, NOTA) showing the ability to get the desired [^18^F]AlF complexes. The authors reported prominent examples of [^18^F]AlF-complexes developed and the various biological targets explored demonstrating the versatility and usefulness of the radioactive synthon. It can be noticed that compared to nucleophilic covalent radiofluorination, most of the time the molar activities obtained are on average significantly lower as most of the time below 50 GBq/µmol but not lower to those obtained for gallium-68 analogs. As the main question is quality of the images to allow nuclear physician to establish diagnosis, the authors reported clinical studies, notably the comparison of [^18^F]AlF-NOTA-octreotide to [^68^Ga]Ga-DOTATE in healthy subjects and patients with NETs or NENs. Further head to head comparison of radiopharmaceuticals comparing [^18^F]AlF-NOTA-octreotide to [^68^Ga]Ga-DOTATE demonstrated a non-inferiority of [^18^F]AlF-NOTA-octreotide to [^68^Ga]Ga-DOTATE and even an advantage with a lower liver uptake. Taken all together, half-life, costs, availability, activities produced, GMP production, images quality, sensitivity, molar activity, these parameters warrants an increase of the uses of [^18^F]AlF in metal-based radiochemistry instead of ^68^Ga in PET radiopharmaceutical development as previously suggested (Fersing et al. 2019).

### ^99m^Tc labeled PSMA inhibitors are still promising!

#### By Junbo Zhang

Prostate-specific membrane antigen (PSMA) has been a promising target for diagnosis and therapy of prostate cancer. Radiolabeled small-molecule PSMA inhibitors have shown potential to be PSMA-targeted radiopharmaceuticals. Because SPECT scanners outnumber PET scanners worldwide and absolute SPECT quantification can also be performed, the development of effective radiolabeled PSMA inhibitors as SPECT tracers for tumor imaging also gained a lot of attention. Technetium-99 m is the best SPECT isotope for the study of novel SPECT radiopharmaceuticals. Thus, to develop easily available ^99m^Tc-labeled PSMA inhibitors for diagnosis of prostate cancer is of great importance. On the development of a suitable ^99m^Tc-labeled probe for PSMA-targeted radio-guided surgery, Tc-99 m-labeled Mas3-y-nal-k(Sub-KuE) (Tc-99 m-PSMA I&S) is a potential candidate. Recently, a single-center prospective study about radiation dosimetry of Tc-99 m-PSMA I&S has been reported (Urbán et al. 2021). They performed a dosimetry study in 4 healthy volunteers and a SPECT/CT imaging study in 10 patients with prostate cancer. They have drawn the following conclusion: the mean effective dose of Tc-99 m-PSMA I&S (0.0052 mSv/MBq) was similar to ^99m^Tc-MDP (0.004 mSv/MBq) and was lower than that of ^68^Ga-PSMA-11(0.0236 mSv/MBq) and ^18^F-PSMA-1007(0.0220 mSv/MBq). Its SPECT/CT images exhibited high tumor/background ratios of primary and metastatic prostate cancer lesions. This study provided preliminary data for translation, but it still needs to include larger trials to verify the ability of ^99m^Tc-PSMA I&S to be a suitable probe for PSMA-targeted radio-guided surgery.

### Automated microfluidic liquid–liquid extraction for radioisotope separation

#### By R. Michael van Dam

Because of the potential for microfluidic devices to enhance speed and performance of chemical processes and reduce instrument size, there has been a long-standing interest in harnessing this technology for many aspects of radiopharmaceutical production.

One area where this has been occurring is in radioisotope production. While solid-phase extraction methods are widely used for the radioisotope purification steps due to the ease of automation using available radiosynthesis equipment, a recent review (Martini et al. 2019) highlighted liquid–liquid extraction (LLE) as another versatile method to purify radioisotopes. The advent of commercially-available LLE technologies has bolstered this approach, making it easier to develop automated separations (Pedersen et al*.* 2018; Martini et al. 2021).

In a nice example of this approach, a microfluidic LLE system was reported to rapidly separate ^99m^Tc from Mo (which is needed for several routes of ^99m^Tc production) with 91% efficiency (Martini et al. 2021). The dissolved target (aqueous phase) and organic solvent (methyl ethyl ketone) flow through a T-junction to generate an alternating series of fluid slugs. As the slugs flow through a residence time loop, ^99m^Tc is selectively extracted from the aqueous phase into the adjacent organic phase. A membrane separator can then collect the organic phase, from which the purified ^99m^Tc can be transferred to saline via a simple cartridge process.

### [^18^F]T‐008, a novel radioprobe for PET imaging of cholesterol 24‐hydroxylase in brain

#### By Ming-Rong Zhang

Cholesterol 24-hydroxylase (CH24H) is a brain-specific enzyme that plays a major role in brain cholesterol homeostasis by changing cholesterol to 24S-hydroxycholesterol. In AD patients, 24S-hydroxycholesterol levels in cerebrospinal fluid were increased compared with healthy subjects. Furthermore, 24S-hydroxycholesterol was shown to regulate various biological functions, including inflammation and oxidative stress. This growing evidence has motivated the development of a PET probe to visualize CH24H and study its function in brain. Recently, a novel radioprobe, 3-[^18^F]fluoroazetidin-1-yl) {1-[4-(4-fluorophenyl)pyrimidin-5-yl]piperidin-4-yl}methanone ([^18^F]T-008) was developed for PET imaging of CH24H in the monkey brain (Koike et al. 2021). [^18^F]T-008 was synthesized by direct ^18^F-fluorination of a tosylated precursor with [^18^F]F^−^. PET study with [^18^F]T-008 on monkey brain showed the highest radioactivity uptake in the striatal regions such as the putamen and caudate (SUV > 4). The rank order of [^18^F]T-008 uptake was striatum > cortical regions > cerebellum, which was consistent with CH24H distribution in the brain. Pre-blocking study with soticlestat, a potent and selective inhibitor for CH24H, reduced the maximum uptake and increased the washout in all brain regions in a dose-dependent manner. Finally, the authors concluded that [^18^F]T-008 is useful for imaging CH24H in the brain and warrants further studies in humans.

### First-in-human evaluation of ^18^F-SynVesT-1, a radioligand for PET imaging of synaptic vesicle glycoprotein 2A

#### By Hongmei Jia

Alterations in synaptic density/plasticity are associated with many neurodegenerative, psychiatric, and addictive disorders. PET imaging of synaptic proteins in vivo will help shed new light on the pathogenesis and progression of synaptopathy in a variety of diseases, and efficacy assessment of disease-modifying therapies. The synaptic vesicle glycoprotein 2A (SV2A), ubiquitously expressed in virtually all synapses, proved to be an excellent target for synaptic imaging. Over the year, a number of ^11^C- and ^18^F-labeled SV2A radioligands have been developed and evaluated. Among these radioligands, [^11^C]UCB-J demonstrates excellent imaging characteristics and has been used to image synaptic density in patients with epilepsy, Alzheimer’s disease, Parkinson’s disease, major depressive disorder, schizophrenia, Huntington’s disease, dementia with Lewy bodies, frontotemporal dementia, progressive supranuclear palsy (PSP), corticobasal syndrome (CBS), cocaine and cannabis use disorders, stroke, and human immunodeficiency virus (HIV). However, the short half-life of ^11^C limits its applications to PET centers with an on-site cyclotron.

Recently, researchers from the Yale PET Center (Naganawa et al. 2021) reported the first-in-human evaluation of [^18^F]SynVesT-1 (also known as [^18^F]SDM-8). In this study, four healthy volunteers participated in a baseline study with [^18^F]SynVesT-1. Four additional subjects were enrolled in a baseline-blocking study with [^18^F]SynVesT-1 and [^11^C]UCB-J using levetiracetam (20 mg/kg) as the blocking drug. The 1-tissue-compartment (1TC) model was judged as the most useful model for quantitative kinetic analysis of [^18^F]SynVesT-1 imaging data. The minimum scan time of 60 min was determined to be sufficient for the stable measurement of regional volume of distribution (*V*_T_). The rank order of regional *V*_T_ and binding potential (*BP*_ND_) was found to be similar between [^18^F]SynVesT-1 and [^11^C]UCB-J. Levetiracetam reduced the uptake of [^18^F]SynVesT-1 in all regions, demonstrating its SV2A binding specificity in vivo. Regional *BP*_ND_ levels of [^18^F]SynVesT-1 were higher than those of [^11^C]UCB-J. The SUVR-1 from 60 to 90 min matched best with 1TC *BP*_ND_ of [^18^F]SynVesT-1 and thus can serve as a surrogate quantitative measurement of specific binding in a short scan time without invasive arterial sampling.

Similar to [^11^C]UCB-J, [^18^F]SynVesT-1 exhibits excellent imaging characteristics with high brain uptake, fast and reversible kinetics, and high specific binding signals. The longer half-life of [^18^F]SynVesT-1 will likely broaden the applications of SV2A imaging to probe synaptic density changes in many neurodegenerative and neuropsychiatric diseases, especially PET imaging studies in larger patient cohorts, and to monitor the effects of emerging therapeutics in clinical trials.

### A clever approach to assess apoptosis by PET imaging

#### By Benjamin Guillet

Apoptosis is an intracellular process that organizes cells to self-destruct in response to various internal or external factors in a stereotypically manner over physiological/physiopathological processes. Apoptosis is the target of a large number of therapeutic strategies and there is a real need for a tool to assess their effectiveness. Molecular imaging would be an option of choice, but till today no imaging agent has been validated. In this noteworthy study, an innovative activated-caspase-3 imaging agents ([^18^F]-C-SNAT4) was developed and evaluated by combining caspase-3 substrate strategy and aggregation of self-assembling nanoparticles to enhance intracellular retention and imaging contrast (Chen et al. 2021a, b). The authors validated caspase-3 targeting in vitro and in vivo in cisplatin-treated sensitive and resistant non-small-cell- lung cancer cells. They showed that tumor uptake of [^18^F]-C-SNAT4 reflects the caspase-3 activity and predicts the chemotherapy outcomes. Additionally, authors reported the effectiveness of [^18^F]-C-SNAT4 TEP imaging in response monitoring to immunotherapy in murine colon tumor model. This observation is crucial since response evaluation to checkpoint inhibitors is essential due to patient high interindividual variability and “pseudoprogression” imaging artefacts on tumor volume or carbohydrate metabolism. Through an original idea, and a rigorous methodology, authors bring a new strategy of apoptosis imaging for oncological, cardiovascular or neurological diseases.

## Conclusions

Trends in radiochemistry and radiopharmacy are highlighted demonstrating the progress in the research field being the scope of EJNMMI Radiopharmacy and Chemistry.

## Data Availability

Datasets mentioned in this article can be found in the cited articles.

## Abbreviations

AD: Alzheimer’s disease
CAF: Cancer-associated fibroblasts
CAR: Chimeric antigen receptor
CT: Computed tomography
ECM: Extracellular matrix
FAP: Fibroblast activation protein
FDG: Fluor-deoxy glucose
FR: Folate receptor
MRI: Magnetic resonance imaging
NET: Neuroendocrine tumor
NSCLC: Non-small cell lung carcinoma
PET: Positron emission tomography
PSMA: Prostate-specific membrane antigen
SPECT: Single photon emission computed tomography
SPS: Spark plasma sintering
SUV: Standardized uptake value
